# SARS-CoV-2 Omicron variant infection affects blood platelets, a comparative analysis with Delta variant

**DOI:** 10.3389/fimmu.2023.1231576

**Published:** 2023-09-27

**Authors:** Cédric Garcia, Baptiste Compagnon, Agnès Ribes, Sophie Voisin, Fanny Vardon-Bounes, Bernard Payrastre

**Affiliations:** ^1^ Inserm UMR1297 and Université Toulouse 3, Institut des Maladies Métaboliques et Cardiovasculaires (I2MC), Toulouse, France; ^2^ Centre Hospitalier Universitaire de Toulouse, Laboratoire d’Hématologie, Toulouse, France; ^3^ Centre Hospitalier Universitaire de Toulouse, Pôle Anesthésie-Réanimation, Toulouse, France

**Keywords:** COVID-19, platelets, omicron variant, LC3B, spike, autophagy

## Abstract

**Introduction:**

In November 2021, the SARS-CoV-2 Omicron variant of concern has emerged and is currently dominating the COVID-19 pandemic over the world. Omicron displays a number of mutations, particularly in the spike protein, leading to specific characteristics including a higher potential for transmission. Although Omicron has caused a significant number of deaths worldwide, it generally induces less severe clinical signs compared to earlier variants. As its impact on blood platelets remains unknown, we investigated platelet behavior in severe patients infected with Omicron in comparison to Delta.

**Methods:**

Clinical and biological characteristics of severe COVID-19 patients infected with the Omicron (n=9) or Delta (n=11) variants were analyzed. Using complementary methods such as flow cytometry, confocal imaging and electron microscopy, we examined platelet activation, responsiveness and phenotype, presence of virus in platelets and induction of selective autophagy. We also explored the direct effect of spike proteins from the Omicron or Delta variants on healthy platelet signaling.

**Results:**

Severe Omicron variant infection resulted in platelet activation and partial desensitization, presence of the virus in platelets and selective autophagy response. The intraplatelet processing of Omicron viral cargo was different from Delta as evidenced by the distribution of spike protein-positive structures near the plasma membrane and the colocalization of spike and Rab7. Moreover, spike proteins from the Omicron or Delta variants alone activated signaling pathways in healthy platelets including phosphorylation of AKT, p38MAPK, LIMK and SPL76 with different kinetics.

**Discussion:**

Although SARS-CoV-2 Omicron has different biological characteristics compared to prior variants, it leads to platelet activation and desensitization as previously observed with the Delta variant. Omicron is also found in platelets from severe patients where it induces selective autophagy, but the mechanisms of intraplatelet processing of Omicron cargo, as part of the innate response, differs from Delta, suggesting that mutations on spike protein modify virus to platelet interactions.

## 1 Introduction

The acute respiratory syndrome coronavirus 2 (SARS-CoV-2), responsible for the coronavirus disease 2019 (COVID-19), undergoes genetic mutations leading to the appearance of new variants (1). Following the Alpha, Beta, Gamma and Delta variants, the SARS-CoV-2 Omicron variant emerged in November 2021 and was swiftly listed as a new variant of concern by the World Health Organization (WHO). It exhibits high transmissibility and has rapidly spread globally, replacing the Delta variant as the dominant strain of SARS-CoV-2 (1). Although the Omicron variant has generally been recognized as less severe compared to Delta (2–4), it has induced a substantial amount of deaths worldwide (https://coronavirus.jhu.edu/map.html). Several sublineages of the Omicron variant are now in circulation.

Blood platelets play a crucial role in normal hemostasis as the initial responders to vascular injury and in thrombosis, particularly atherothrombosis (5). These effector cells also have important functions in inflammation and host defense (5–9). It is well-established that platelets, thrombosis and inflammation are intricately interconnected in numerous pathologies, including infectious diseases (7–9). Platelet activation during severe COVID-19 is thought to contribute to the thromboembolic events and to worsen inflammatory response culminating in acute respiratory distress syndrome (ARDS). Moderate thrombocytopenia, as well as alterations in platelet activation, correlate with disease severity and mortality (10–15). Furthermore, several studies have reported platelet hyperactivation and their interaction with monocytes (16–20). Electron microscopy in *post-mortem* samples has revealed platelet-rich thrombi at sites of infected pulmonary endothelium (21).

Despite the low presence of angiotensin-converting enzyme 2 (ACE2), the best documented receptor for SARS-CoV-2, in platelets, it has been demonstrated that platelets from COVID-19 patients contain viral particles (12, 20, 22, 23) and can internalize SARS-CoV-2 *in vitro* through various pathways (12, 20). In addition to SARS-CoV-2, other viruses such as dengue and influenza have been shown to infect megakaryocytes and be present in platelets (24, 25). SARS-CoV-2 infection was found to modify megakaryocytes and platelets transcriptome, triggering an antiviral response phenotype (17, 26).

Omicron variant, characterized by several mutations in the spike protein, including the receptor-binding domain, exhibits increased transmissibility potential and generally induces milder disease severity (1, 27, 28). Contrasting with previous variants, the entry of Omicron into host cells seems to be independent of the transmembrane serine protease 2 (TMPRSS2) (28, 29), suggesting differences in viral phenotype, including the mode of internalization.

Whether the Omicron variant affects blood platelets remains poorly known. Here, platelet parameters including activation and sensitivity were investigated as well as the presence of virus and the autophagy response in severe COVID-19 patients infected with different variants.

## 2 Methods

### 2.1 Study design

Patients were prospectively included in a prospective observational study in a medico-surgical intensive care unit (ICU) from April 12^th^, 2021 to May 25^th^, 2022. Eligible patients were hospitalized for hypoxemic pneumonia caused by SARS-CoV-2 infection. Variants identification was performed through complete sequencing of the spike gene. Ethical approval was obtained from the Comité de protection des personnes du sud-ouest et outre-mer (CPP2020-04-042a/2020-A00972-37/20.04.08.64705) and all patients gave their consent to participate, in accordance with the Declaration of Helsinki. Patients with malignant blood diseases or hemostatic disorders, pregnant women, minors or individuals under protective measures were excluded. Baseline characteristics including hemostasis parameters (obtained from the Laboratory of haematology, Rangueil Academic Hospital of Toulouse, France), and outcomes were recorded upon admission to the ICU. Blood was collected 3.8 ± 3.2 and 3.2 ± 1.4 days after ICU admission and 15.6 ± 10.1 and 9.1 ± 4.4 days after symptom onset for patients infected with the Delta and the Omicron variants, respectively. SARS-CoV-2 serology was assessed using the SARS-CoV-2 multi-antigen serology module (5 antigens analyzed simultaneously: S1 RDB, Nucleocapsid, S2 subunit, S1 subunit, Spike) allowing detection of IgG antibodies in plasma by Simple Western capillary electrophoresis (22). Seropositivity and seronegativity was determined according to the cut-offs provided by the manufacturer.

Platelet function tests were not realized if acetyl salicylic acid or corticosteroids were taken within the 10 days prior inclusion.

The recruitment of healthy donors was approved by the Toulouse Hospital Bio-Resources biobank, registered with the Ministry of Higher Education and Research (DC2016-2804). These donors were tested negative for PCR at inclusion, and had not taken antiplatelets or steroids within the 10 days before blood sampling. The cohort consisted of 50% females, with median age of 38 years (interquartile range, 26-69).

### 2.2 Materials

Collagen-related peptide (CRP) was obtained from Prof. R. Farndale Laboratory (Cambridge, UK), and the collagen reagent Horm^®^ (equine) suspension was purchased from Takeda (St. Peter Strasse, Austria). Anti-CD62P conjugated FITC, anti-CD63 conjugated FITC, anti-PAC1 conjugated FITC, antibodies were obtained from BD Biosciences and the anti-GPVI antibody was obtained from Biocytex (Marseille, France). The SARS-CoV-2 (2019-nCoV) Spike S1(D614G)-His recombinant protein and SARS-CoV-2 BQ.1.1 (Omicron) Spike S1+S2 trimer proteins were sourced from SinoBiological (Beijing, China). All other reagents were purchased from Sigma Aldrich (Saint-Louis, USA). **Supplementary Materials** provide further details regarding the sources and references of the antibodies.

### 2.3 Blood sampling and plasma preparation

Whole blood samples were collected using Vacutainer tubes containing 3.2% citrate (Becton Dickinson). Platelet-rich plasma (PRP), platelet-poor plasma (PPP), and washed platelets were obtained through differential centrifugation as described previously (22). PPP, used for measuring soluble markers, was stored at -80°C. Washed platelets were resuspended in modified HEPES Tyrode’s buffer with a pH of 7.4 and containing 2 mM CaCl_2_ at 2x10^8^ platelets/mL for confocal microscopy and western-blot analysis.

### 2.4 Light transmission platelet aggregation tests

Platelet aggregation was monitored with a turbidimetric method (SD Medical, STAGO, France) following previously described protocols (22). PRP was stimulated with various agonists (CRP 9 μg/mL, thrombin receptor agonist peptide (TRAP) 50 μM, collagen 0.75 μg/mL, thromboxane A2 analog U46619 1 μM) for 10 minutes under continuous stirring conditions (1000 revolution min^-1^) at 37°C using siliconized glass cuvettes. The thrombosoft 1.6 software (SD Medical) was used to determine the maximal platelet aggregation percentage.

### 2.5 Flow cytometry analysis

Flow cytometry was performed using FACSVERSE (BD Biosciences), and FASCsuites software (BD Biosciences) was used for data analysis. Platelet granule secretion was evaluated by measuring surface expression of CD62P and CD63, which serve as markers for α and δ granules, respectively. The activation status of GPIIbIIIa was assessed using PAC1 antibody. PRP was incubated in non-shaking conditions with TRAP at a concentration of 50 μM, U46619 at 5 µM, or CRP at 9 µg/mL for a duration of 10 minutes. Subsequently, PRP was incubated with the appropriate antibodies at room temperature for 15 minutes. After the addition of phosphate buffer saline (PBS), the samples were subjected to cytometer analysis. The results were expressed as median fluorescence intensity (MFI). The number of copy of GPIa, GPIb, GPIIIa, and GPVI on the platelet surface was quantified using a specific commercial kit (Platelet GP Screen, Biocytex) following the manufacturer’s instructions (22).

### 2.6 ELISA assay, capillary western assay and western blotting

The proinflammatory cytokines interleukin-6 (IL-6), interleukin-8 (IL-8), and TNF alpha were quantified in human plasma using micro-ELISA using ELLA technology (ProteinSimple). Soluble P-selectin, and soluble CD40L were measured in human plasma using ELISA kits (Mybiosources, San Diego, USA; Life Technologies SAS, Thermo Fischer, Courtaboeuf, France) following the manufacturer’s instructions. The capillary western blot assay was performed using all reagents provided by ProteinSimple, except for the primary antibodies, which were obtained separately. Platelet homogenates were prepared and analyzed as previously described (30).

For western blotting, washed platelets from patients or controls were subjected to analysis using specific antibodies according to standard procedures.

### 2.7 Ultrastructural analysis by transmission electron microscopy

Platelets in 1 mL of PRP were fixed for 1 hour at 37°C with 2.5% glutaraldehyde in 19 ml of 0.1 M Sorensen phosphate buffer (pH 7.4). TEM was performed and data analyzed as previously described (22).

### 2.8 Confocal microscopy immunocytochemistry

Platelets (2.10^8^/mL) were prepared and processed as described previously (22). Confocal images were acquired using a 63X objective and an LSM900 confocal laser microscope (Carl Zeiss). Super-resolution imaging was performed using the Airyscan module. For the quantification of colocalization, the FIDJI software with the JACOP plug-in was used to calculate Pearson’s correlation coefficient and Manders’ M1 coefficient (22).

### 2.9 Phosphoflow cytometry

Washed platelets resuspended in modified HEPES Tyrode’s buffer containing 2 mM CaCl2 (2x10^8^ platelets/mL) were incubated in polypropylene tubes with or without (basal) spike proteins from Delta or Omicron variants (5 µg/mL final concentration), for 1, 10, and 30 minutes at 37°C without stirring. Platelets were then analyzed as we recently described (31). FACS analysis was conducted in a single run per primary antibody (up to 16 barcoding possibilities) using a BD LSR Fortessa™ flow cytometer (BD Biosciences). Platelets were gated on their scatter properties and 500,000 events per tube were analyzed for MFI (5-10 minutes per tube).

### 2.10 Statistical analysis

Statistical analysis was performed using nonparametric tests, including the Mann-Whitney test to compare patients, the Wilcoxon test for experiments with recombinant proteins and the Fisher test for TEM analysis. P<0.05 was statistically significant. GraphPad Prism (La Jolla, CA, USA) was used to realize the figures.

## 3 Results

### 3.1 Characteristics of the patients

Twenty patients admitted to the ICU from April 12^th^, 2021 to May 25^th^, 2022 infected with SARS-CoV-2 Delta variant (n=11) or Omicron variant (n=9), as assessed by RT-PCR and spike gene sequencing, were enrolled as well as 10 healthy donors. The biological and clinical data of the patients are provided in **Supplementary Table 1**. There was no differences between the groups in terms of patient history and biological data. The SAPS II severity score tended to be higher in patients in the Delta group compared to the Omicron group. Regarding the ARDS severity (PaO2/FiO2 ratio), the Omicron group patients had a lower median ratio than those in the Delta group [89 (11–131) *vs.* 125 (68–235) p<0.03] at admission (**Table 1**). Patients in the Delta group were more ventilated and had longer durations of ICU and hospital stay compared to the Omicron group, but their mortality rates were similar (**Supplementary Table 1**). Consistent with previous findings for the Delta variant (22), the median concentrations of pro-inflammatory cytokines (IL-6, IL-8 and TNF alpha) were significantly higher in patients with Omicron compared to controls (**Table 1**).

Table 1Platelet activation markers and reactivity in patients with SARS-CoV-2 Omicron Variant.

**Table:** 

	Healthy (n=10)	Omicron (n=4, 2 seronegative, 2 seropositive)	p Value
Membrane glycoproteins – mean (range)			
GPIIb/IIIa (Number of copies/platelets)	52425 (42323–66002)	35334 (26648–50785)	0.038*
GPIb (Number of copies/platelets)	33205 (25536-42629)	28086 (25469-33592)	0.087
GPIa/IIa (Number of copies/platelets)	5206 (4077-7461)	3858 (3194-4539)	0.034*
GPVI (Number of copies/platelets)	4898 (3323-6603)	3115 (2501-4277)	0.014*
Secretion of P-selectin – mean (range)			
Basal (MFI)	12 (4-29)	13 (8-23)	0.681
TRAP 50µM (MFI)	400 (140-911)	285 (152-451)	0.198
U46619 5µM (MFI)	583 (358-873)	265 (93-459)	0.014*
CRP 9µg/mL (MFI)	728 (612-873)	422 (250-656)	0.036*
Secretion of CD63 – mean (range)			
Basal (MFI)	12 (7-28)	15 (9-24)	0.368
TRAP 50µM (MFI)	179 (129-254)	113 (81-134)	0.002**
U46619 5µM (MFI)	206 (132-284)	103 (21-162)	0.036*
CRP 9µg/mL (MFI)	301 (235-407)	187 (114-255)	0.027*
Activation of GpIIb/IIIa (PAC1) – mean (range)			
Basal (MFI)	5 (2-17)	3 (1-6)	0.179
TRAP 50µM (MFI)	202 (64-361)	63 (3-234)	0.086
U46619 5µM (MFI)	273 (103-379)	77 (2-244)	0.039*
CRP 9µg/mL (MFI)	343 (188-413)	191 (42-377)	0.122

**Table:** 

	Healthy (n=10)	Omicron (n=9)	p Value
Plasma soluble platelet activation markers – mean (range)			
Soluble P-Selectin (ng/mL)	54.8 (42.8-64.1)	107.8 (43.9-183.0)	0.04*
Soluble CD40L (ng/mL)	0.8 (0.1-1.7)	3.3 (0.5-5.9)	0.03*
Soluble GPVI (ng/mL)	9.1 (6.4-15.1)	52.3 (11.7-190.5)	0.0001***
Plasma cytokines – mean (range)			
Interleukin 6 (pg/mL)	1.9 (0.7-3.52)	212.8 (3.1-782.0)	0.038*
Interleukin 8 (pg/mL)	4.0 (3.7-4.46)	26.1 (7.4-51.7)	0.001**
TNF alpha (pg/mL)	7.7 (6.2-10.7)	15.4 (9.1-30.0)	0.009**

P value Significance denoted as *p <.05, **p <.01, and ***p <.0001 represents the comparison of healthy donors vs. Omicron patients based on the nonparametric Mann-Whitney test.

### 3.2 Platelet characteristics in patients with severe hypoxemic pneumonia due to omicron variant

Surface expression of several platelet glycoproteins indicated that, while the level of GPIb remained unchanged, the expression of GPIaIIa, GPIIbIIIa (α_IIb_β3) and GPVI significantly decreased in platelets from severe patients infected with Omicron, suggesting the occurrence of a shedding process (**Table 1**). Moreover, electron microscopy analysis revealed signs of prior platelet activation. Indeed, platelets from severe COVID-19 patients infected with Omicron had a reduction in the number of microtubules associated with more pseudopodia (**Supplementary Figure 1**). Soluble GPVI (sGPVI), soluble P-selectin (sCD62P) and soluble CD40 ligand (sCD40L) were significantly increased in the plasma of patients infected with the Omicron variant compared to controls (**Table 1**). The increase in these specific soluble platelet activation markers was not related to the seropositive or seronegative status of the patients **(Supplementary Figure 2**) and confirmed a shedding process.

At rest, the surface expression of α and dense granules secretion markers P-selectin (CD62P) and CD63, respectively, as well as the activation of αIIbβ3 integrin, did not show significant differences in controls and in patients infected with the Omicron variant (**Table 1**). However, platelet stimulation by TRAP, U46619 or CRP efficiently increased the membrane expression of CD62P and CD63 as well as αIIbβ3 integrin activation, albeit with a lesser intensity in platelets from patients compared to controls. These results indicate that platelets from severe COVID-19 patients infected with the Omicron variant exhibit signs of preactivation and are partially hyporesponsive, similar to what has been shown previously in severe COVID-19 patients infected with the Delta variant (22, 32).

### 3.3 Omicron variant induces a platelet viral infection response phenotype

**Figure 1:**
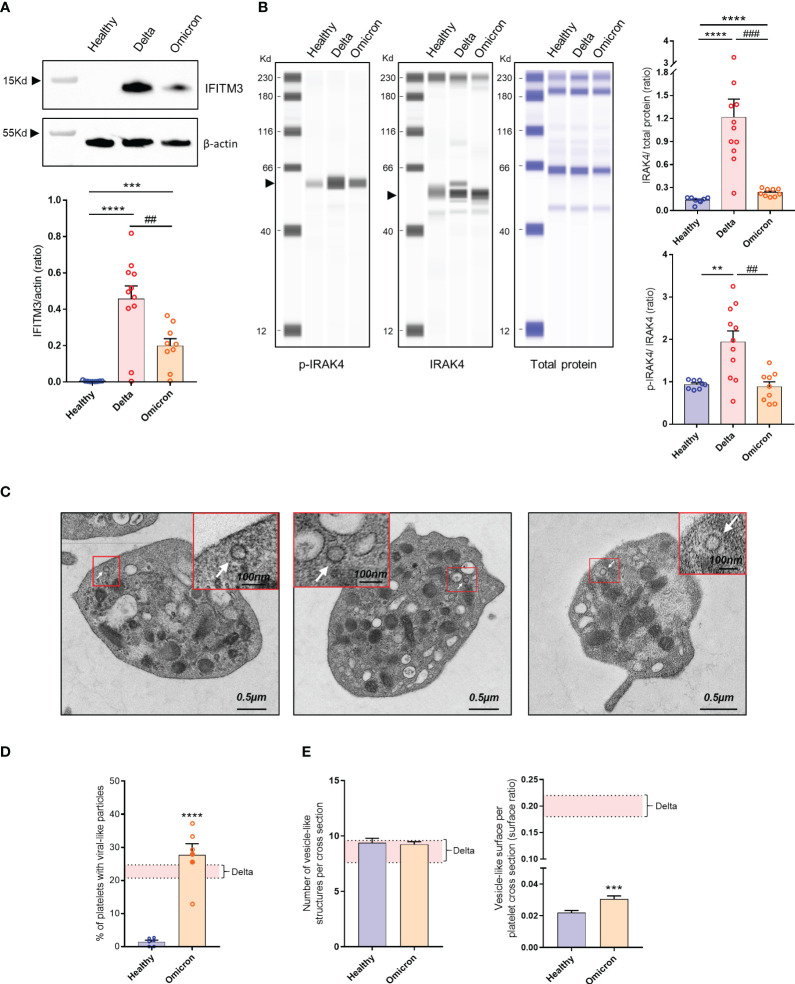
Platelet phenotype in severe COVID-19 patients infected with the Omicron variant. Representative Western blotting analysis of IFITM3 levels in platelets from 8 healthy donors (Healthy), 11 severe COVID-19 patients with the Delta variant (Delta), and 9 severe COVID-19 patients with the Omicron variant (Omicron) is shown **(A)**. As an index of the purity of the washed platelet preparation, the amount of remaining leukocytes was 1 leukocyte/1000 platelets. The quantification of the IFITM3/actin ratio is shown. ## p <.01 represents the comparison of Delta patients *vs.* Omicron patients; *** p<.001 and ****p <.0001 represent the comparison of healthy donors *vs.* Delta or Omicron patients, based on the nonparametric Mann-Whitney test. Capillary Western Assay analysis of phospho- and total IRAK4 in platelets from 8 healthy donors (Healthy), 11 severe COVID-19 patients with the Delta variant (Delta), and 9 severe COVID-19 patients with the Omicron variant (Omicron) is shown **(B)**. The quantification of the phospho-IRAK4 (p-IRAK4)/Total protein ratio and the IRAK4/Total protein ratio is shown in graphs. Significance denoted as ## p<.01 and ### p<.0001 represents the comparison of Delta patients *vs.* Omicron patients. Significance denoted as ***p <.001 and ****p <.0001 represents the comparison of healthy donors *vs.* Delta or Omicron patients based on the nonparametric Mann-Whitney test. Detection of SARS-CoV-2 in platelets from severe COVID-19 patients with the Omicron variant was performed using transmission electron micrographs revealing the presence of characteristic crown-like structures resembling SARS-CoV-2 particles **(C)**. Images shown are representative of those obtained from the 6 patients with the Omicron variant (2 seronegative, 4 seropositive) analyzed by TEM. Viral-like particles (white arrows) were identified based on specific criteria, including the size of the viral particle (100 nm ± 20%) as described (22). Quantitative analysis was performed on transmission electron micrographs (186 from n = 6 healthy donors [31 per donor], and 486 from n = 6 patients with the Omicron variant [80 per patient]). The percentage of platelets from healthy controls and patients with the Omicron variant containing viral-like particles were quantified **(D)**. Results are mean ± SEM; each circle represents an individual sample (full circle: seropositive, empty circle: seronegative). Significance denoted as **** p<.0001 represents the comparison of healthy donors *vs.* Omicron patients based on the nonparametric Mann-Whitney test. The number of vesicle-like structures per cross-section **(E)**, and the vesicle-like surface/platelet cross-section surface ratio were quantified using electron micrographs of complete platelet cross-sections. Significance denoted as ***p <.001 represents the comparison of healthy donors *vs.* Omicron patients based on the nonparametric Mann-Whitney test. The pink areas in the graphs represents the mean +/- SEM we previously found in platelets from severe patients with the Delta variant (22).

Infection has been shown to modify the transcriptome of megakaryocytes and platelets, leading to the induction of an antiviral response phenotype (26). For example, interferon (IFN)-induced transmembrane protein 3 (IFITM3) known to inhibit viral replication, is upregulated in megakaryocytes and platelets from patients infected with the Delta variant (17). Interestingly, platelets from patients infected with Omicron also exhibited IFITM3 expression, although to a lesser extent compared to those infected with Delta (**Figure 1A**). Additionally, as markers of Toll-like receptors (TLR) activation, interleukin-1 receptor-associated kinase 4 (IRAK4) was upregulated and phosphorylated in platelets from Delta variant-infected patients, while it showed weak upregulation and poor phosphorylation in platelets from Omicron-infected patients (**Figure 1B**).

Platelets from six severe Omicron-infected patients were analyzed by TEM. Characteristic crown-like structures exhibiting a size consistent with SARS-CoV-2 particles were observed (**Figure 1C**). These viral-like particles were detected in all tested patients but not controls (**Figure 1D**). The percentage of platelets containing viral-like particles was 27.7 ± 3.4%, slightly higher than the percentage we previously reported for the Delta variant (22).

**Figure 2:**
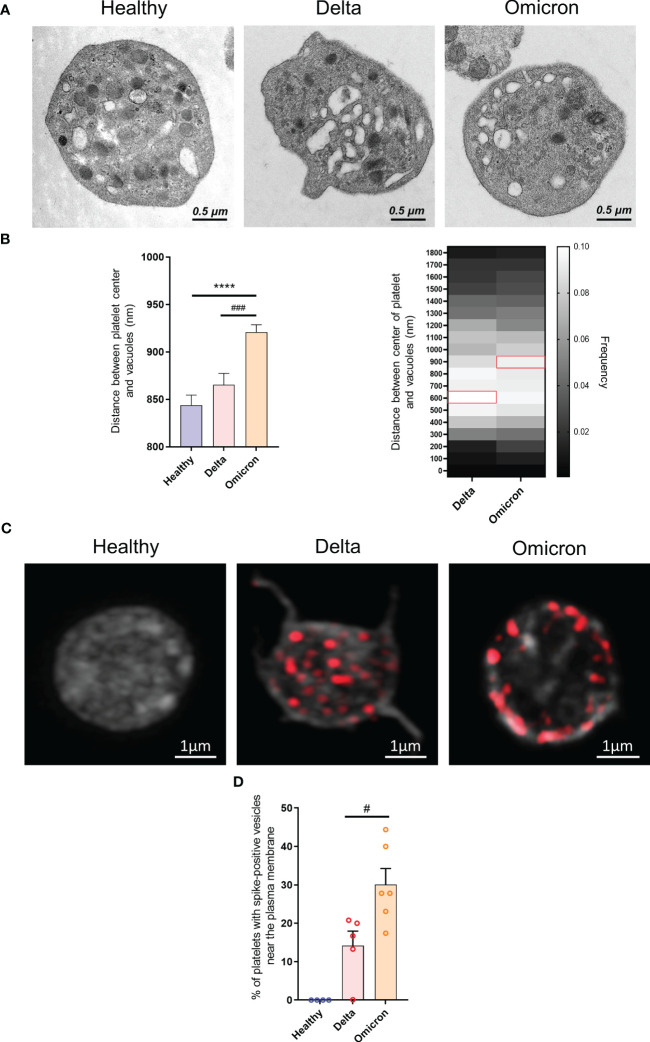
Ultrastructure of platelets in severe COVID-19 patients infected with the Omicron variant. Transmission electron micrographs of platelets from patients infected with the Delta or the Omicron variants and controls **(A)** were imported into QuPath software and regions of interest were annotated. Once the selection and annotation were complete, the entire platelet was displayed in green while the vacuoles were shown in red. The pixel size was determined by using the scale bar provided on the electron micrograph. The brush tool was used to draw and annotate the regions of interest. The software determined the position of the centroids allowing to measure the distance between the center of the platelet and the vesicle centroids. The position of 1683 vesicles for the Delta variant (300 platelets from 10 patients), 3705 vesicles for the Omicron variant (486 platelets from 6 patients) and 1790 vesicles for controls (129 platelets from 4 healthy donors) were determined **(B)**. The presence of SARS-CoV-2 spike protein within platelets from Delta or Omicron infected patients was analyzed by super-resolution confocal microscopy **(C)**, and the positions of spike-positive vesicles within platelets was determined. The percentage of platelets exhibiting labeling at the periphery of the cytoplasmic membrane was quantified **(D)**. Significance denoted as #p <.05 and ###p <.0001 represents the comparison of Delta *vs.* Omicron patients based on the nonparametric Mann-Whitney test. Significance denoted as **** p <.0001 represents the comparison of healthy donors vs. delta patients based on the nonparametric Mann-Whitney test.

A distinguishing feature of platelets from severe Delta-infected subjects was the existence of enlarged vesicles that were distinct from the open canalicular system (OCS) (22). Electron microcopy analysis of complete platelet cross-sections showed that platelets from Omicron-infected patients exhibited enlarged vesicles that were smaller than those seen in Delta infection (**Figure 1E**). Another difference was the distribution of these vesicles, which were more peripheral and closer to the plasma membrane in platelets from Omicron-infected patients (**Figures 2A, B**). Interestingly, super-resolution confocal microscopy also indicated that spike-positive structures were localized near the plasma membrane in platelets from Omicron-infected patients (**Figures 2C, D**). These findings suggest a distinct mechanism of viral entry or processing in platelets.

### 3.4 Omicron infection increases platelet autophagy structures and colocalization of Rab7 and spike

**Figure 3:**
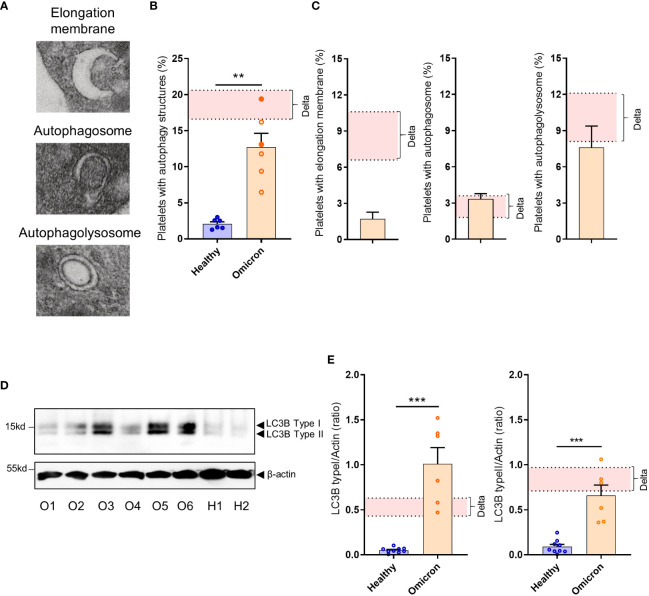
Platelets from severe COVID-19 patients infected with the Omicron variant have autophagic structures. Representative TEM sections of platelets from severe COVID-19 patients infected with the Omicron variant highlighting structures typical of elongation membrane (EM), autophagosome (AP), and autophagolysosome-like (AL) are shown **(A)**. The percentage of platelets containing autophagy structures is presented. Each circle represents an individual (full circle: seropositive, empty circle: seronegative) and results are mean ± SEM **(B)** along with the frequency of occurrence of the different types of structures **(C)** in platelets from 6 healthy donors and 6 severe COVID-19 patients with the Omicron variant. Representative Western blotting analysis of LC3B-I and LC3B-II levels in platelets from 6 severe COVID-19 patients with the Omicron variant (O1 to O6) and 9 healthy donors (H1, H2) are shown **(D)**. The exposure time was adjusted in order to efficiently distinguish the LC3B type I and type II. The quantification of the LC3B-I/actin ratio and LC3B-II/actin ratio for healthy donors (n=9), and patients with the Omicron variant (n=6) is shown **(E)**. Significance denoted as **p <.01 and ***p <.001 represents the comparison of healthy donors *vs.* Omicron patients, based on the nonparametric Mann-Whitney test. The pink areas in the graphs represents the mean +/- SEM we previously found in platelets from severe patients with the Delta variant (22).

We have previously demonstrated that platelets can activate an intrinsic antiviral defense mechanism involving the degradation of viral material through autophagosomes and autophagolysosomes (22). TEM analysis revealed that SARS-CoV-2 Omicron infection also induces selective autophagy in platelets from infected patients, characterized by an increased number of autophagosomes and autophagolysosomes (**Figures 3A–C**). However, compared to Delta, Omicron showed limited efficiency in inducing the formation of elongation membranes (phagophores) (**Figure 3C**) suggesting a different mechanism in the initiation of autophagy. The expression level of LC3B type I and LC3B type II, markers of autophagosomes and autolysosomes, were significantly elevated in platelets from Omicron-infected subjects compared to controls (**Figures 3D, E**).

**Figure 4:**
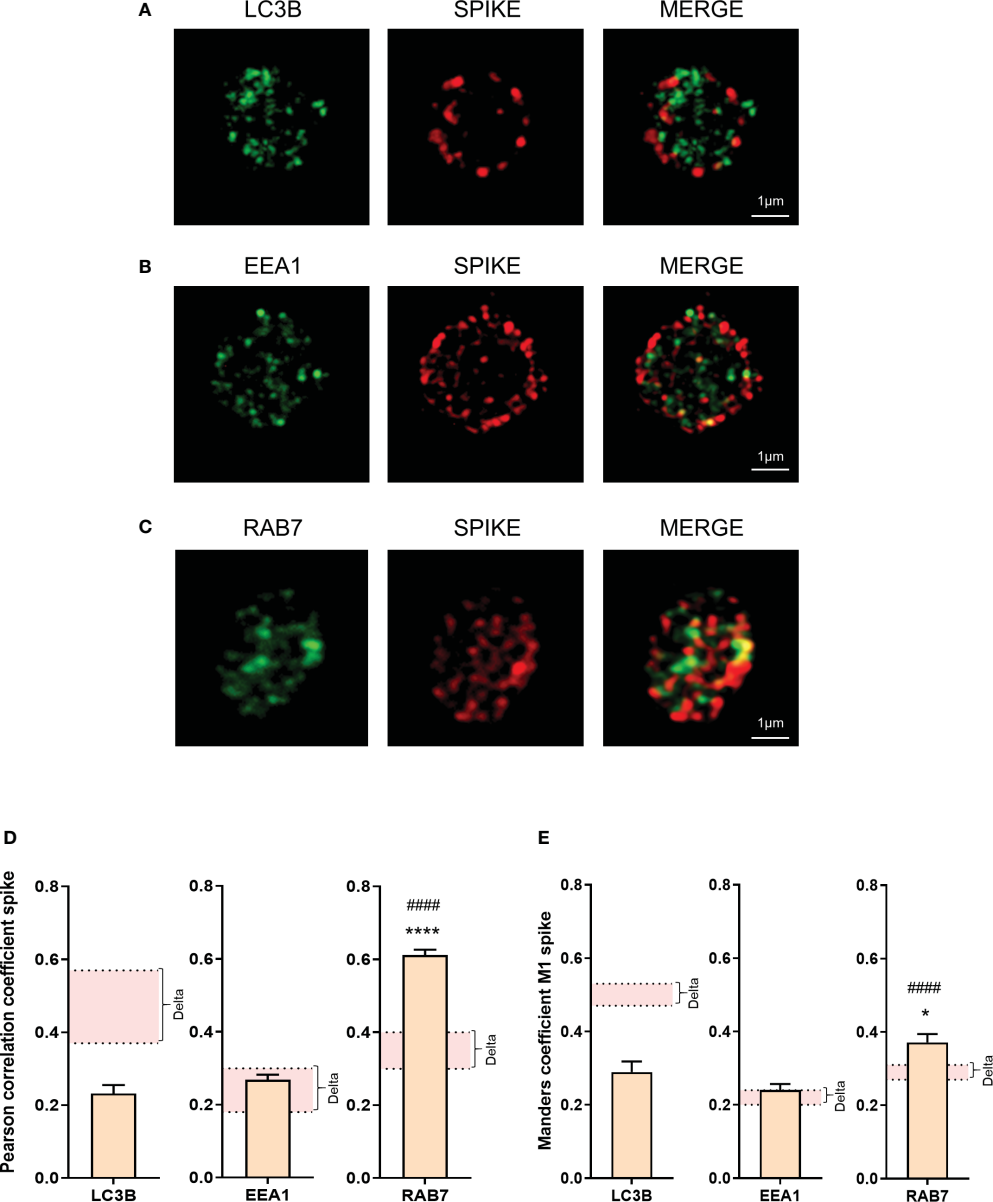
Omicron spike protein subcellular distribution in platelets from severe COVID-19 patients. The intraplatelet localization of the spike protein was investigated using immunofluorescence and super-resolution confocal microscopy with the Airyscan module. A specific anti-spike antibody was used. Its colocalization with LC3B, a marker of autophagosomes **(A)**, EEA1, a marker of early endosomes **(B)**, and Rab7, a marker of late endosomes **(C)**, was analyzed. Representative images from 6 different patients with severe COVID-19 are shown. Quantification was performed by analyzing 30 to 40 platelets from each of the 6 patients with the Omicron variant (4 seronegative, 2 seropositive). Pearson’s correlation coefficient and Manders’ coefficient calculations are presented **(D, E)**. Significance denoted as *p <.05 and ****p <.001 represents the comparison between the colocalization of spike protein with LC3B *vs.* the colocalization of spike protein with Rab7. Significance denoted as #### p <.001 represents the comparison between the colocalization of spike protein with EEA1 and the colocalization of spike protein with Rab7. The pink areas in the graphs corresponds to the mean +/- SEM we previously found in the Delta variant (22).

Super-resolution microscopy showed a weaker colocalization between the spike protein and LC3B in platelets from Omicron-infected patients, compared to Delta. The colocalization between spike protein and EEA1 (early endosome marker) was relatively low and similar, while the colocalization between spike protein and Rab7 (a late endosome marker) was significantly increased in platelets from Omicron-infected patients (**Figures 4A–E**). Compared to Delta variant, involving LC3B-associated phagocytosis and selective autophagy (22), the strong colocalization between spike protein and Rab7 suggests the existence of endosomal pathway-derived elements in platelets from Omicron-infected subjects. Furthermore, spike proteins from Omicron or Delta variants were separately added to platelets from healthy donors. Subcellular localization analysis indicated that, compared to the spike protein from the Delta variant, the spike protein carrying mutations present in Omicron exhibited a significantly higher colocalization with Rab7 and a lower colocalization with LC3B (**Supplementary Figures 3A–D**).

### 3.5 Effect of Omicron and Delta spike proteins on platelet signaling

**Figure 5:**
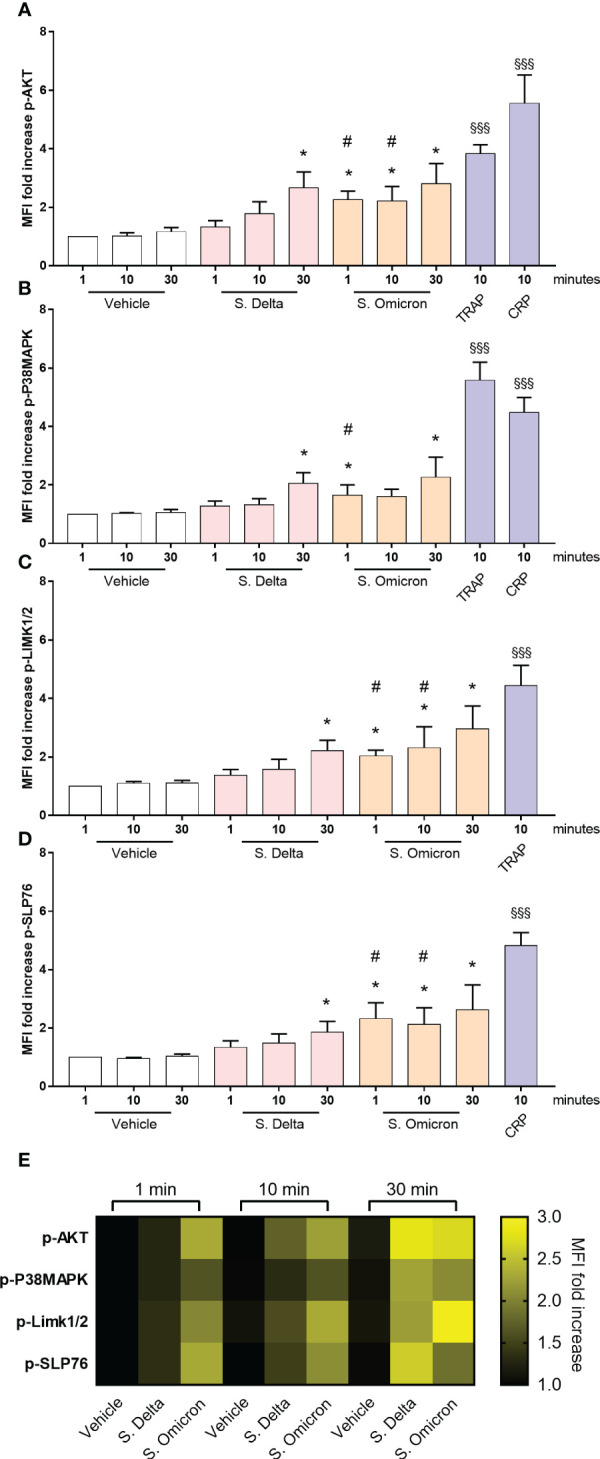
Healthy platelet signaling pathways activated by Omicron and Delta spike proteins. Washed platelets from 7 healthy donors were incubated in the presence or absence of 5µg/mL of the Delta or Omicron variants spike proteins for 1, 10 and 30 minutes at 37°C without shaking. Phosphorylation of the signaling proteins AKT **(A)**, P38MAPK **(B)**, LIMK1/2 **(C)**, and SLP76 **(D)** was monitored over time using phosphoflow cytometry (31). As a comparison, washed platelets were also stimulated with 50 µM TRAP or 9 µg/ml CRP for 10 minutes at 37°C without shaking. Results are presented as the MFI ratio of spike protein-stimulated platelets to resting platelets (mean ± SEM, n= 7). **(E)** The heatmap summarizes the evolution of phosphorylation intensity for each protein as a function of platelet stimulation time. Statistical significance is indicated as *p<.05, according to the nonparametric Wilcoxon test for comparison of vehicle *vs.* Delta or Omicron spike proteins. Additionally, #p<.05 represents the comparison of Delta *vs.* Omicron spike protein using the nonparametric Wilcoxon test. Significance denoted as §§§p <.001 indicates comparison of vehicle *vs.* agonists, according to the nonparametric Wilcoxon test.

Subsequently, we investigated whether the differences in the intraplatelet processing of the two variants would correspond to distinct signaling potential of their respective spike proteins. Washed control platelets were incubated for 1, 10 and 30 minutes with the two spike proteins in non-shaking conditions, and the phosphorylation of signaling intermediates was assessed by phosphoflow cytometry (31). Both spike proteins induced a significant increase in AKT, P38MAP-kinase, LIM-kinase and SLP76 phosphorylation (**Figure 5**). However, the signal intensity was lower compared to that elicited by CRP or TRAP. Interestingly, the Delta spike protein exhibited a slow activation of signaling pathways (30 minutes), whereas the Omicron spike protein triggered a much faster response (within 1 minute), suggesting a distinct mode of action associated with the acquisition of Omicron spike protein mutations.

## 4 Discussion

The involvement of platelets in severe COVID-19 has been established in various studies and patient cohorts worldwide (12, 13, 33, 34). However, SARS-CoV-2 is continuously evolving through mutations and the resulting variants have different degrees of contagiousness and virulence (1, 2, 4, 35). Currently, the Omicron variant and its sub-variants are the predominant circulating strains worldwide (1, 36). With these mutations, the Omicron variant has demonstrated increased efficiency in replicating in the bronchi, thereby enhancing its transmission through higher viral load expelled during coughing (37). Additionally, a decrease in replication capacity in the lungs (29, 37) and a loss of ability to fuse pneumocytes during replication (28, 38, 39) have been observed, which mitigates lung damages (40) and contribute to the reduced severity of the disease. The decreased pathogenicity of Omicron resulted in fewer ICU admissions. Nevertheless, its high infectivity has led to a significant number of infections and subsequent hospitalizations (41).

In our cohort of patients, with similar comorbidities, we observed that the overall severity, as assessed by the clinical-biological SAPS II score, was more unfavorable for the Delta variant. Our study indicates that patients infected with Delta had a worse outcome compared to those infected with omicron. This is reflected in multiple organ failures, higher incidence of mechanical ventilation and longer ICU and hospital stays in the Delta patients group. However, it is noteworthy that the criteria for ICU admission were broader during the period of Delta variant patients, which may have contributed to higher average PaO2/FiO2 ratios at the time of admission. Nonetheless, these patients remained severe, as evidenced by higher SAPS II scores and longer lengths of stay compared to the Omicron variant.

Omicron infected patients exhibited signs of platelet preactivation, such as elevated levels of plasma soluble activation markers (sGPVI, sCD40L, sCD62P), decreased number of microtubules and increased pseudopodia. Shedding of platelet surface markers including GPIaIIa, α_IIb_β3 and GPVI, related to proteolytic cleavage during platelet activation, was also observed. These preactivation platelet phenotypes have already been reported in severe subjects infected with the Delta variant (12, 16, 18, 19, 22). Furthermore, platelets from severe patients infected with Omicron exhibited decreased α_IIb_β3 integrin activation following stimulation with some physiological agonists, along with a decrease in granules secretion. This partial desensitization suggests that, preactivated platelets display a hyporesponsive phenotype, which has also been observed in severe Delta infection (22, 32, 42). Thus, Omicron variant mutations have not eliminated the platelet phenotype previously described in severe patients infected with ancestral strains. The origin of the platelet phenotype in COVID-19 is still unclear and may be attributed to the severity of the disease, characterized by high inflammatory conditions and presence of neutrophil extracellular traps (43). However, a direct interaction between SARS-CoV-2 and megakaryocytes as well as platelets may occur (23, 34). Platelets are known to rapidly respond to pathogen infections (7–9). We found that the Omicron variant induced the expression of the antiviral immune effector gene IFITM3 in platelets, albeit to a lesser extent compared to Delta-infected patients. This significant upregulation of IFITM3 in platelets, driven by viral infection, indicates the implication of megakaryocytes and platelets to the antiviral response triggered by Omicron. Upregulation of IFITM3 in platelets from patients infected with Delta variant as well as dengue and influenza viruses has been reported (17, 26). Of note, a multi-omics approach suggests that Omicron infection triggers a strong platelet antiviral immune signature (44). Interestingly, recent data suggest a potential link between IFITM3 and platelet hyperreactivity under inflammatory stress conditions (45).

We also observed that, while IRAK4, a reflection of TLR activation, was upregulated and phosphorylated in platelets from patients infected with Delta, only weak upregulation and phosphorylation were observed in platelets from subjects with Omicron. This result suggests that, as previously observed for HIV-1 pseudovirions (46), infection with the Delta variant triggers platelet TLR activation while the Omicron variant does not induce the same level of platelet TLR activation.

Viral RNA sequencing and TEM analysis have shown the presence of the Delta variant inside patients platelets (12, 16, 17, 20, 22, 23). We show using TEM that severe patients infected with Omicron have virus-like particles in 27.7 ± 3.4% of platelets. This finding is consistent with previous data on platelets from patients infected with Delta variant (12, 22). In our small cohort of patients, the elevation of platelet activation markers and the presence of virus in platelets were observed both in seropositive and seronegative patients. Interestingly, super-resolution confocal microscopy revealed differential distribution of the spike protein in platelets according to the type of variant. The Omicron spike protein was observed near the plasma membrane, suggesting that both variants have different mechanisms of entry and/or processing into platelets. The subcellular distribution of Omicron spike-positive vesicles around the plasma membrane and their colocalization with the small GTPase Rab7, an important regulator of late endosome maturation and function, strongly suggest that the viral material traffics through the endocytic pathway. This finding is consistent with studies demonstrating that the entry of Omicron preferentially uses the endocytic cell entry pathway compared to previous variants (37, 39). *In vitro*, the absence of TMPRSS2 in host cells redirects SARS-CoV-2 Delta variant toward an endocytic pathway involving Rab7-positive late endocytic vesicles (47). Due to the loss of interaction with TMPRSS2, the Omicron variant primarily utilizes this pathway (37). However, as Dengue and Severe fever with thrombocytopenia syndrome viruses (24, 48), SARS-CoV-2 may replicate in platelets (23). Although this is yet uncertain (49), we cannot exclude that Rab7-spike colocalization could reflect the presence of secretory lysosomes containing spike as a cargo (50).

It is noteworthy that *in vitro* experiments with recombinant spike protein carrying Omicron mutations alone demonstrated its internalization in healthy platelets, with colocalization with Rab7. Moreover, spike proteins from the Delta and Omicron variants alone induced the phosphorylation of AKT, P38MAP-kinase, LIM-kinase and SLP76 in healthy platelets, but with different time courses. The Delta spike protein activated signaling pathways slower compared to the Omicron spike protein, suggesting that the acquired mutations modify the interaction of the spike protein with platelets. In real life, one may suggest that spike-antibody immune complexes may also contribute to platelet activation via FcγRIIA.

Our data also suggest the coexistence and interconnection of the endolysosomal and autophagic pathways in severe patient’s platelets. Similar to platelets from subjects infected with the Delta variant (22), we observed that the Omicron variant triggers selective autophagy in patient platelets, as evidenced by an increased number of autophagosomes and autophagolysosomes. The expression of LC3B type I and LC3 type II were also elevated. A difference between the two variants is the decreased number of elongation membranes in the case of Omicron. These structures are highly transitory and one may suggest that the Delta variant slow down this step by using proteins such as Nsp6 that can interfere with the elongation membrane process (51). Mutations on Nsp6 in the Omicron variant (52) may rescue a fast elongation membrane process difficult to capture by TEM.

The autophagy process initiated by the different variants may be important to degrade viral material present in the cytosol, including proteins like nucleocapsid released after virus entry or material up-taken from plasma by platelets. In the case of Delta variant, autophagy and LC3-associated phagosomes may contribute to degrade viral material after fusion with lysosomes. In contrast, the Omicron variant seems to enter through the endosomal pathway and coexist with the autophagic process to eliminate viral material. It is important to note that the virus entering megakaryocytes and platelets has likely developed strategies to counteract its elimination by these pathways (52).

Therefore, platelets, as a prominent component of blood, may participate in the elimination of the Omicron variant or its components. Supporting this possibility, a recent report suggests a correlation between platelet counts and Omicron clearance efficiency (53). Additionally, it is plausible to suggest that platelets, and megakaryocytes (23), may contribute to the dissemination of the virus, which first affects the lungs and then spreads via the blood stream, infecting various organs and tissues. A recent study has shown that Omicron can indeed spread to multiple tissue locations after primary infection (54). The mechanisms by which the virus enters platelets and megakaryocytes remain unclear, but besides a low ACE2 expression, several candidates for SARS-CoV-2 receptors have been proposed (34), including GPIb (55) and CD147 (49). CLEC2 was also recently added as a direct spike sensor (56). Moreover, FcγRIIA on platelets and megakaryocytes may interact with the Fc fragment of immunoglobulin G-virion complexes contributing to internalization. Thus, platelets and megakaryocytes can employ various receptors and certainly different intracellular mechanisms to sense and respond to SARS-CoV-2 infection.

Considering that SARS-CoV-2 is an evolving and mutating virus, it is important to explore how platelets respond to different variants in COVID-19 patients, not only in terms of thrombotic potential but also in their antiviral and immune functions. This understanding is crucial for elucidating the interaction between platelets and the virus and proposing potential therapeutic strategies. In this study, we demonstrate that platelets from severe patients infected with Omicron are activated, partially desensitized, and internalize a significant proportion of viral particles. Despite the decreased pathogenicity of Omicron, the platelet phenotype closely resembles that observed in subjects contaminated with Delta. However, we provide evidence that platelets have evolved different mechanisms to respond to SARS-CoV-2 mutations and ensure interaction with the virus.

## Data availability statement

The original contributions presented in the study are included in the article/**Supplementary Material**. Further inquiries can be directed to the corresponding authors.

## Ethics statement

The studies involving humans were approved by Comité de protection des personnes du sud-ouest et outre-mer (CPP2020-04-042a/2020-A00972-37/20.04.08.64705). The studies were conducted in accordance with the local legislation and institutional requirements. The participants provided their written informed consent to participate in this study.

## Author contributions

Conception-design of the work: BP, CG, and FV-B. Patient inclusion, clinical management duties, and ethical approvals: FV-B, BC, AR, and SV. Experimental design and execution of the majority of the experiments: CG and BC. Data compilation, analysis, and interpretation: CG, AR, SV, FV-B, and BP. Manuscript writing: BP, CG, and FV-B. Manuscript review and critical editing: all authors. All authors contributed to the article and approved the submitted version.
