# Supplementary material for: SARS-CoV-2 Omicron variant infection affects blood platelets, a comparative analysis with Delta variant
Source: Front Immunol. 2023 Sep 27;14:1231576. doi: 10.3389/fimmu.2023.1231576 (PMC10565689; doi:10.3389/fimmu.2023.1231576)
Supplement: Supplementary file 1 [file DataSheet_1.pdf]

Supplementary material

Antibodies references

Antibody	Isotype	Provider	Reference
SARS Coronavirus Spike	Rabbit	Abcam	ab272504
Phospho-IRAK4 (Thr345/Ser346) (D6D7) mAb	Rabbit	Cell Signaling Technology	#11927
IRAK4 Antibody	Rabbit	Cell Signaling Technology	#4363
IFITM3 (D8E8G) XP® mAb	Rabbit	Cell Signaling Technology	#59212
Phospho-Akt (Ser473) (D9E) XP®	Rabbit	Cell Signaling Technology	#4060
Phospho-p38 MAPK (Thr180/Tyr182)	Rabbit	Cell Signaling Technology	#4511
Anti-SLP76 (phospho Y145) antibody	Rabbit	Abcam	ab75829
LC3B (E5Q2K) Mouse mAb	Mouse	Cell Signaling Technology	#83506
LC3B (E7X4S) XP® Rabbit mAb	Rabbit	Cell Signaling Technology	#43566
Alexa Fluor™ 594 Phalloidin		Thermofisher	A12381
EEA1 (E9Q6G)	Mouse	Cell Signaling Technology	#48453
Rab7 (E9O7E)	Mouse	Cell Signaling Technology	#9367
Goat anti-Rabbit IgG (H+L) Secondary Antibody, Alexa Fluor 647		Thermofisher	# A-21244
Goat anti-Mouse IgG (H+L), Secondary Antibody, Alexa Fluor™ 488		Thermofisher	# A-28175

Supplementary Table 1: Characteristics of the patients with severe COVID-19.

	Delta Variant (n=11)	Omicron Variant (n=9)	p Value
**Demographic characteristics**			
Age –median (range)	64 (40-72)	62 (35-80)	
Male sex – no. (%)	8 (72)	8 (88)	
BMI –median (range)	32.4 (26.4-52.2)	30.5 (23.4-38.6)	
SAPS II –median (range)	53.0 (22.0-67.0)	32.0 (22.0-60.0)	
Baseline SOFA –median (range)	6.0 (3.0-12.0)	4.0 (2.0-16.0)	
Vaccination status – no. (%)	3 (27.3)	4 (44.4)	
SARS-CoV-2 positive serology – no. (%)	8 (72.7)	4 (44.4)	
**Co-existing conditions** – no. (%)			
Hypertension	6 (54.0)	1 (11.0)	
Diabetes mellitus	0 (0.0)	2 (18.0)	
Dyslipidemia	0 (0.0)	1 (11.0)	
Previous myocardial infarction	1 (9.0)	1 (11.0)	
Chronic respiratory disease	0 (0.0)	1 (11.0)	
Obesity	8 (72.0)	6 (67.0)	
Chronic cardiac disease	0 (0.0)	0 (0.0)	
Chronic liver disease	0 (0.0)	1 (11.0)	
Chronic kidney disease	0 (0.0)	1 (11.0)	
Thrombo-embolic disease	1 (9.0)	2 (18.0)	
Prior antiplatelet agent	0 (0.0)	0 (0.0)	
Prior anticoagulation	1 (9.0)	1 (11.0)	
Coinfection (bacteria or fungus)	7 (63.0)	5 (55.0)	
**Biology (at inclusion)** –median (range)			
CRP (mg/mL)	44.5 (1.3-230.0)	130.3 (14.1-347.0)	
Leukocytes (G/L)	10.6 (2.7-22.1)	8.4 (3.7-63.7)	
Lymphocytes (G/L)	0.8 (0.0-1.0)	1.0 (0.4-61.4)	
Neutrophils (G/L)	10.2 (2.4-18.3)	6.3 (2.0-12.0)	
Monocytes (G/L)	0.6 (0.2-1.0)	0.3 (0.2-0.7)	
Platelet count (G/L)	212 (78-394)	255 (135-331)	
MPV (fL)	9.9 (8.8-13.7)	10.3 (9.2-12.4)	
D-dimers (ng/mL)	770 (610-4000)	1190 (470-4000)	
Fibrinogen (g/L)	4.7 (1.9-8.4)	6.5 (1.8-10.7)	
**Therapeutics in ICU and Outcomes**, median (range) or no. (%)			
Ratio PaO2/FiO2	125 (68-235)	89 (11-131)	0.03*
High Flow Oxygen - no. (%)	9 (81.0)	7 (78.0)	
Mechanical ventilation - no. (%)	11 (100.0)	5 (55.0)	0.02*
Duration of mechanical ventilation (days)	14 (6-74)	17 (15-29)	
Dexamethasone at inclusion – no. (%)	9 (82)	9 (100)	
Tocilizumab – no. (%)	6 (55)	6 (67)	
Venous thromboprophylaxis	11 (100)	9 (100)	
Antibiotics at inclusion – no. (%)			
Cefepime	2 (19)	2 (11)	
Piperacillin-tazobactam	8 (73)	7 (78)	

Meropenem	0 (0)	1 (11)	
Vasopressors – no. (%)	7 (64)	4 (44)	
Continuous Renal Replacement Therapy – no. (%)	2 (19)	1 (11)	
ICU LOS (days)	35 (9-383)	15 (6-59)	0.01*
Hospital LOS (days) – median (range)	39 (21-408)	25 (7-59)	0.03*
Death – no. (%)	5 (45)	4 (44)	

P value Significance denoted as*p <.05, represents the comparison of Delta vs. Omicron patients based on the nonparametric Mann-Whitney test for Ratio PaCo2/FiO2, ICU, and hospital LOS, Fisher's test was used for the comparison of mechanical ventilation.
BMI: Body Mass Index, SAPS II: Simplified Acute Physiology Score, SOFA: Sepsis-related Organ Failure Assessment, COPD: Chronic Obstructive Pulmonary Disease, CRP: C-Reactive Protein, MPV: Mean Platelet Volume, ICU: Intensive Care Unit, LOS: Lenght Of Stay

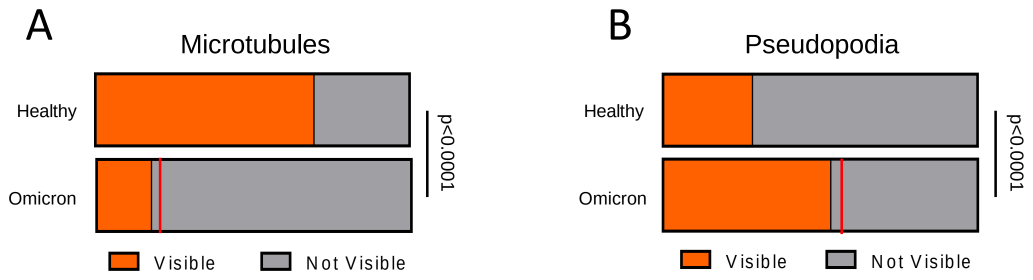

Figure S1: Platelet from severe COVID-19 patients infected with Omicron have ultrastructural signs of activation

Quantitative analysis was performed on transmission electron micrographs. A total of 186 micrographs were analyzed from 6 healthy donors (31 micrographs each), and 486 micrographs were analyzed from 6 patients with the Omicron variant (80 micrographs each). The percentage of platelets exhibiting or not microtubules (A) and pseudopods (B) was quantified from cross-sections. These parameters were selected to reflect the level of platelet activation. The red bar indicates the percentage previously found for platelets from severe patients with Delta variant (22). The Fischer test was used for statistical analysis.

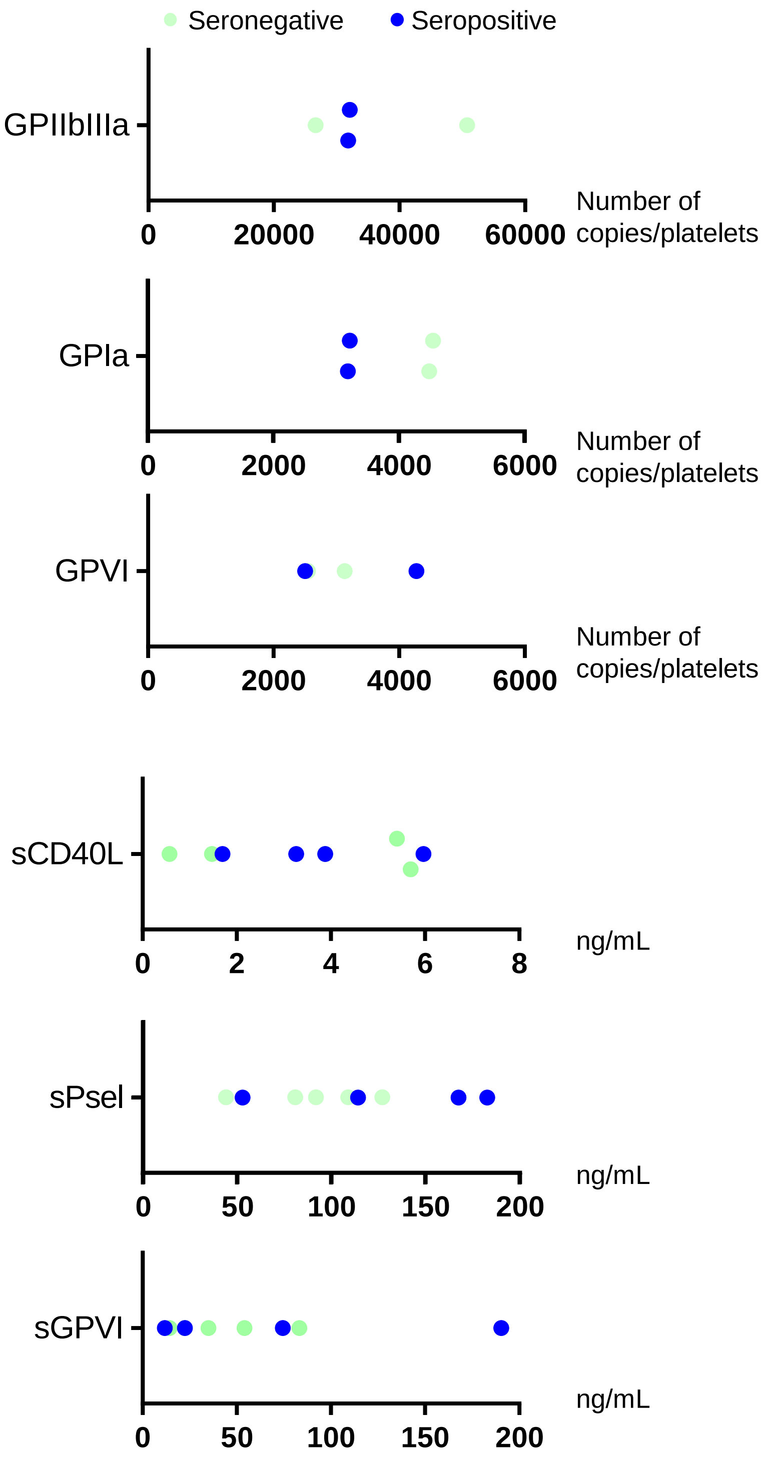

Figure S2: Impact of serology on platelet glycoproteins and soluble platelet activation markers

Results shown in Table 1 are presented here as individual patients separated according to their seronegative (green) or seropositive (blue) statues.

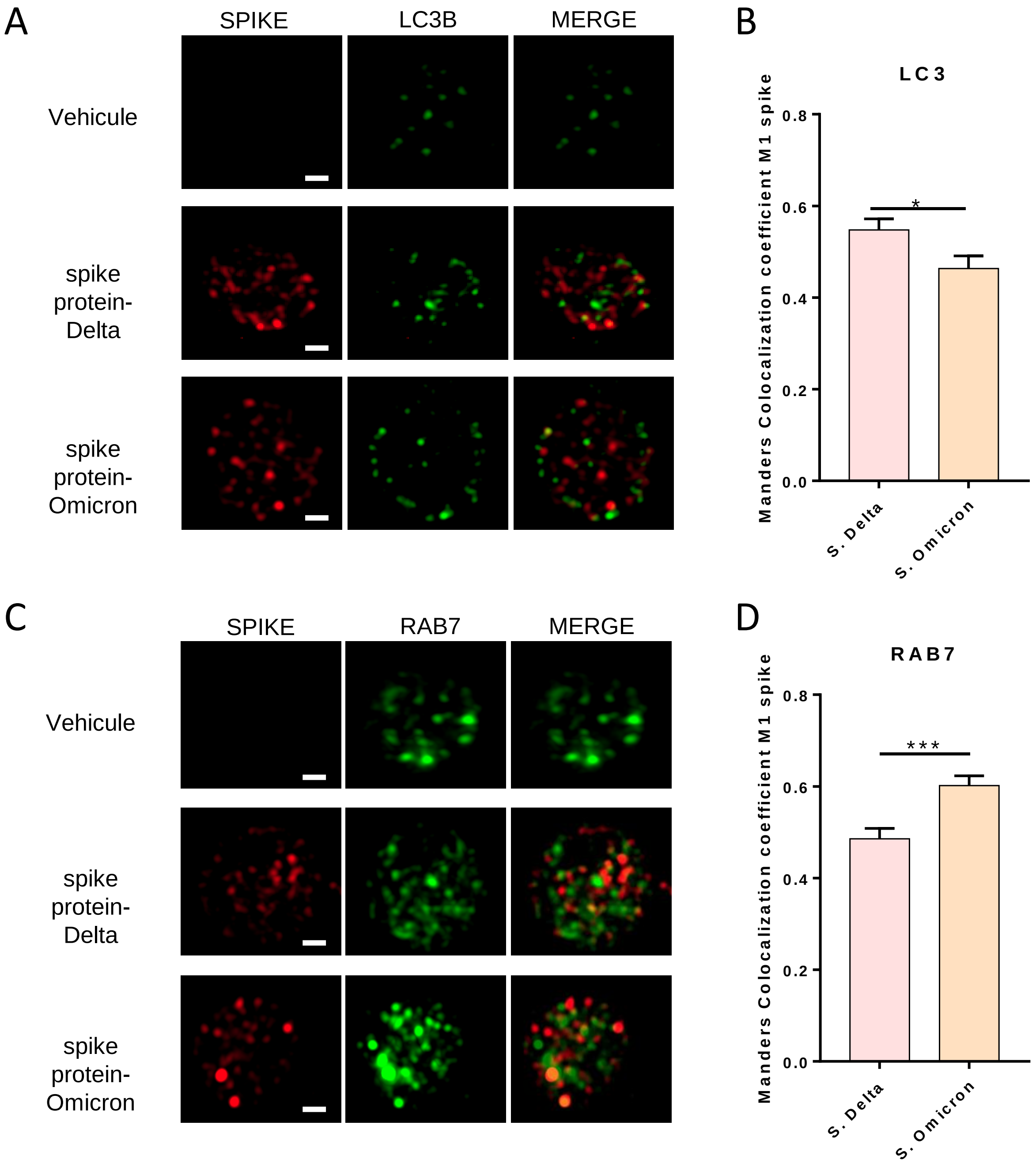

Figure S3: Localisation of Omicron and Delta spike proteins following incubation with healthy platelets.

Washed healthy platelets were incubated with or without 5µg/mL of spike protein from Delta or Omicron variants, for 30 minutes at 37°C without shaking. After fixation and permeabilization, the intraplatelet localization of the spike proteins was examined using immunofluorescence and super-resolution confocal microscopy with the Airyscan module. A specific anti-spike antibody was utilized for this purpose. (A) Representative images from 4 healthy donors, for each condition, are shown. The colocalization of the spike proteins (S. Delta and S. Omicron) with LC3B was quantified from 30 platelets for each condition and the Manders coefficient was calculated (B). The same experimental approach was performed for Rab7, a late endosome marker (C and D). Results are presented as mean ± SEM and statistical analysis was performed using the nonparametric Mann-Whitney test, with *$p < .05$ and ***$p < .001$ indicating significance. The scales bar represents 1 µm.
